# Supplementary material for: Deletion of wfs1 Impairs Oligodendrocyte Precursor Cells Dorsal Distribution and Myelination Through the wfs1-hmgcs1 Axis in Zebrafish
Source: Biology (Basel). 2026 Aug 16;15(16):1402. doi: 10.3390/biology15161402 (PMC13510151; doi:10.3390/biology15161402)
Supplement: Supplementary file 1 [file biology-15-01402-s001.zip › File S1-Transcriptome sequencing data and differentially expressed genes (DEGs) in wfs1-- zebrafish at 5 dpf/2.KEGG/wfs1KOvsWT/GSEA/WT/CARDIAC_MUSCLE_CONTRACTION/CARDIAC_MUSCLE_CONTRACTION(DRE04260).html]

Details for gene set CARDIAC\_MUSCLE\_CONTRACTION(DRE04260)[GSEA]

|  || Dataset | deg.sample |
| Phenotype | sample.cls |
| Upregulated in class | WT |
| GeneSet | CARDIAC\_MUSCLE\_CONTRACTION(DRE04260) |
| Enrichment Score (ES) | -0.24658374 |
| Normalized Enrichment Score (NES) | -1.0408622 |
| Nominal p-value | 0.4274354 |
| FDR q-value | 0.5764769 |
| FWER p-Value | 1.0 |
Table: GSEA Results Summary

  

Fig 1: Enrichment plot: CARDIAC\_MUSCLE\_CONTRACTION(DRE04260)      
 Profile of the Running ES Score & Positions of GeneSet Members on the Rank Ordered List

  

| PROBE | DESCRIPTION (from dataset) | GENE SYMBOL | GENE\_TITLE | RANK IN GENE LIST | RANK METRIC SCORE | RUNNING ES | CORE ENRICHMENT || 1 | 556638\_2 | slc9a6b |  |  | 498 | 2.997 | 0.0127 | No |
| 2 | 100535196 | cacna2d3a |  |  | 702 | 2.590 | 0.0309 | No |
| 3 | 447942 | cox6a2 |  |  | 746 | 2.477 | 0.0530 | No |
| 4 | 677750 | myl2b |  |  | 901 | 2.226 | 0.0693 | No |
| 5 | 559015\_1 | cacnb3b |  |  | 1252 | 1.850 | 0.0758 | No |
| 6 | 114457\_1 | atp1b2b |  |  | 1641 | 1.574 | 0.0785 | No |
| 7 | 393940 | atp2a2a |  |  | 1653 | 1.567 | 0.0930 | No |
| 8 | 100124599 | smyhc2 |  |  | 1918 | 1.402 | 0.0980 | No |
| 9 | 794522 | si:ch211-139a5.9 |  |  | 1956 | 1.387 | 0.1100 | No |
| 10 | 100037358 | zgc:163073 |  |  | 2005 | 1.366 | 0.1214 | No |
| 11 | 393793 | uqcrc1 |  |  | 2224 | 1.277 | 0.1266 | No |
| 12 | 751638 | cox5b2 |  |  | 2518 | 1.168 | 0.1285 | No |
| 13 | 557499 | slc8a4b |  |  | 2704 | 1.110 | 0.1332 | No |
| 14 | 64616 | atp1a1b |  |  | 2763 | 1.093 | 0.1417 | No |
| 15 | 553296 | hrc |  |  | 3174 | 0.998 | 0.1382 | No |
| 16 | 494489 | atp2a1l |  |  | 3699 | 0.892 | 0.1302 | No |
| 17 | 64609\_1 | atp1a2a |  |  | 3837 | 0.861 | 0.1340 | No |
| 18 | 570210 | zgc:86709 |  |  | 3984 | 0.834 | 0.1373 | No |
| 19 | 569480 | slc8a2a |  |  | 4033 | 0.822 | 0.1436 | No |
| 20 | 403311 | cacna1db |  |  | 4082 | 0.814 | 0.1498 | No |
| 21 | 797554 | cacng4a |  |  | 4426 | 0.757 | 0.1462 | No |
| 22 | 245703 | atp1a1a.5 |  |  | 4968 | 0.686 | 0.1356 | No |
| 23 | 554532 | slc9a7 |  |  | 5087 | 0.669 | 0.1383 | No |
| 24 | 565158\_1 | cacnb3a |  |  | 5266 | 0.650 | 0.1388 | No |
| 25 | 64616\_1 | atp1a1b |  |  | 5478 | 0.636 | 0.1382 | No |
| 26 | 568759 | cacna2d2b |  |  | 5927 | 0.595 | 0.1297 | No |
| 27 | 64609 | atp1a2a |  |  | 6196 | 0.568 | 0.1266 | No |
| 28 | 64615 | atp1a1a.4 |  |  | 6471 | 0.540 | 0.1231 | No |
| 29 | 570210\_2 | zgc:86709 |  |  | 6553 | 0.532 | 0.1256 | No |
| 30 | 568463\_2 | ryr2b |  |  | 6731 | 0.516 | 0.1249 | No |
| 31 | 556638 | slc9a6b |  |  | 6829 | 0.507 | 0.1267 | No |
| 32 | 560561 | slc8a3 |  |  | 6854 | 0.504 | 0.1307 | No |
| 33 | 568225 | cacna2d4a |  |  | 6934 | 0.498 | 0.1329 | No |
| 34 | 559964\_2 | cacna1fb |  |  | 7527 | 0.453 | 0.1186 | No |
| 35 | 101882457 | cacna2d1 |  |  | 8073 | 0.417 | 0.1053 | No |
| 36 | 100331116 | cacng5b |  |  | 8521 | 0.401 | 0.0950 | No |
| 37 | 100333135 | si:ch1073-325m22.2 |  |  | 8533 | 0.400 | 0.0985 | No |
| 38 | 793708 | cacna1fa |  |  | 8992 | 0.368 | 0.0876 | No |
| 39 | 100330915 | si:dkey-85n7.8 |  |  | 9079 | 0.361 | 0.0883 | No |
| 40 | 100329748\_1 | LOC100329748 |  |  | 9141 | 0.357 | 0.0897 | No |
| 41 | 795135 | slc9a1 |  |  | 9326 | 0.345 | 0.0872 | No |
| 42 | 571193 | cacng1b |  |  | 9443 | 0.337 | 0.0867 | No |
| 43 | 405791 | cacna1sb |  |  | 9453 | 0.337 | 0.0897 | No |
| 44 | 541376 | cacna2d2a |  |  | 9515 | 0.333 | 0.0909 | No |
| 45 | 100150428\_1 | cacna2d4b |  |  | 9544 | 0.330 | 0.0931 | No |
| 46 | 797199\_1 | slc8a1b |  |  | 9790 | 0.316 | 0.0884 | No |
| 47 | 64611 | atp1a3b |  |  | 9850 | 0.313 | 0.0895 | No |
| 48 | 322013 | cacng1a |  |  | 10196 | 0.291 | 0.0814 | No |
| 49 | 568463 | ryr2b |  |  | 10274 | 0.286 | 0.0817 | No |
| 50 | 64267 | atp1b1a |  |  | 10535 | 0.271 | 0.0761 | No |
| 51 | 100150428 | cacna2d4b |  |  | 10848 | 0.252 | 0.0686 | No |
| 52 | 100148645 | cacna2d3 |  |  | 10863 | 0.251 | 0.0706 | No |
| 53 | 563476 | cox7b |  |  | 10950 | 0.247 | 0.0702 | No |
| 54 | 100034580 | cacng6a |  |  | 11524 | 0.215 | 0.0542 | No |
| 55 | 140541 | COX3 |  |  | 11660 | 0.208 | 0.0519 | No |
| 56 | 100537244 | myl2a |  |  | 11772 | 0.202 | 0.0503 | No |
| 57 | 562422 | cacnb4a |  |  | 13149 | 0.133 | 0.0082 | No |
| 58 | 100003278 | cacng7b |  |  | 13204 | 0.130 | 0.0077 | No |
| 59 | 767664\_1 | cox7a1 |  |  | 13979 | 0.094 | -0.0158 | No |
| 60 | 393614 | cacng2a |  |  | 14251 | 0.080 | -0.0236 | No |
| 61 | 140539 | COX1 |  |  | 15120 | 0.041 | -0.0506 | No |
| 62 | 64272 | atp1b3b |  |  | 15172 | 0.038 | -0.0518 | No |
| 63 | 64269 | atp1b2a |  |  | 15271 | 0.034 | -0.0546 | No |
| 64 | 100034540 | cacng7a |  |  | 15278 | 0.033 | -0.0545 | No |
| 65 | 140540 | COX2 |  |  | 15750 | 0.009 | -0.0692 | No |
| 66 | 140512 | CYTB |  |  | 16039 | -0.003 | -0.0783 | No |
| 67 | 170581 | cacna1c |  |  | 16091 | -0.005 | -0.0798 | No |
| 68 | 567438\_1 | cacng3a |  |  | 16145 | -0.007 | -0.0815 | No |
| 69 | 562640 | si:dkey-28b4.8 |  |  | 16374 | -0.013 | -0.0885 | No |
| 70 | 100329813\_1 | LOC100329813 |  |  | 16377 | -0.013 | -0.0885 | No |
| 71 | 568463\_1 | ryr2b |  |  | 16528 | -0.018 | -0.0930 | No |
| 72 | 100329813 | LOC100329813 |  |  | 16620 | -0.021 | -0.0957 | No |
| 73 | 554103 | cox7a2a |  |  | 16682 | -0.022 | -0.0974 | No |
| 74 | 321552 | smyhc1 |  |  | 16751 | -0.024 | -0.0993 | No |
| 75 | 64610 | atp1a3a |  |  | 17262 | -0.043 | -0.1150 | No |
| 76 | 792983\_1 | slc9a6a |  |  | 17340 | -0.047 | -0.1170 | No |
| 77 | 792983 | slc9a6a |  |  | 17474 | -0.053 | -0.1207 | No |
| 78 | 678560 | cacnb4b |  |  | 17849 | -0.069 | -0.1318 | No |
| 79 | 797199 | slc8a1b |  |  | 17863 | -0.070 | -0.1315 | No |
| 80 | 795492 | cacnb2a |  |  | 17901 | -0.072 | -0.1320 | No |
| 81 | 561434 | cacna2d1a |  |  | 17908 | -0.072 | -0.1315 | No |
| 82 | 568671 | atp2a3 |  |  | 18013 | -0.076 | -0.1341 | No |
| 83 | 436925 | cacnb1 |  |  | 18557 | -0.101 | -0.1502 | No |
| 84 | 64273 | atp1b1b |  |  | 18730 | -0.110 | -0.1546 | No |
| 85 | 569770 | cox8b |  |  | 19098 | -0.126 | -0.1650 | No |
| 86 | 321552\_1 | smyhc1 |  |  | 19284 | -0.135 | -0.1696 | No |
| 87 | 101885761 | slc9a1a |  |  | 19374 | -0.138 | -0.1711 | No |
| 88 | 353248 | tnnt2c |  |  | 19676 | -0.151 | -0.1791 | No |
| 89 | 260440 | atp2a1 |  |  | 20102 | -0.171 | -0.1909 | No |
| 90 | 100037383 | atp1b4 |  |  | 20122 | -0.172 | -0.1899 | No |
| 91 | 100003329 | cacng8b |  |  | 20276 | -0.178 | -0.1930 | No |
| 92 | 559236 | unm\_hu7910 |  |  | 20586 | -0.193 | -0.2009 | No |
| 93 | 554101 | cox5aa |  |  | 20691 | -0.198 | -0.2023 | No |
| 94 | 406359 | uqcrfs1 |  |  | 20779 | -0.203 | -0.2031 | No |
| 95 | 415164 | zgc:86725 |  |  | 20902 | -0.209 | -0.2050 | No |
| 96 | 30468 | atp1b3a |  |  | 21050 | -0.218 | -0.2076 | No |
| 97 | 335774 | cox6a1 |  |  | 21080 | -0.220 | -0.2064 | No |
| 98 | 564379 | cyc1 |  |  | 21143 | -0.223 | -0.2062 | No |
| 99 | 566723 | uqcrh |  |  | 21287 | -0.230 | -0.2086 | No |
| 100 | 393478 | cox6b2 |  |  | 21307 | -0.231 | -0.2070 | No |
| 101 | 100535363 | cox7a2b |  |  | 21548 | -0.243 | -0.2122 | No |
| 102 | 406437 | tpm4a |  |  | 21677 | -0.250 | -0.2139 | No |
| 103 | 574000 | tnnt2d |  |  | 22059 | -0.270 | -0.2233 | No |
| 104 | 324244 | tpm4b |  |  | 22140 | -0.275 | -0.2232 | No |
| 105 | 322549 | uqcrc2b |  |  | 22169 | -0.276 | -0.2215 | No |
| 106 | 436930 | uqcrc2a |  |  | 22306 | -0.284 | -0.2231 | No |
| 107 | 436968 | cox6b1 |  |  | 22427 | -0.291 | -0.2241 | No |
| 108 | 553489 | slc8a1a |  |  | 22439 | -0.292 | -0.2217 | No |
| 109 | 568265 | atp2a2b |  |  | 22467 | -0.294 | -0.2198 | No |
| 110 | 408256 | actc1a |  |  | 22616 | -0.303 | -0.2216 | No |
| 111 | 336118 | cox7c |  |  | 22818 | -0.314 | -0.2249 | No |
| 112 | 550428 | cacng8a |  |  | 22994 | -0.327 | -0.2273 | No |
| 113 | 567212 | slc8a2b |  |  | 23204 | -0.341 | -0.2307 | No |
| 114 | 767664 | cox7a1 |  |  | 23291 | -0.347 | -0.2301 | No |
| 115 | 326975 | cox4i1 |  |  | 23518 | -0.362 | -0.2338 | No |
| 116 | 326962 | cox5ab |  |  | 23797 | -0.380 | -0.2390 | No |
| 117 | 402880 | cox4i1l |  |  | 23812 | -0.381 | -0.2358 | No |
| 118 | 415209 | tpm2 |  |  | 23895 | -0.386 | -0.2347 | No |
| 119 | 556703\_1 | tnnt2e |  |  | 23899 | -0.386 | -0.2312 | No |
| 120 | 570683 | cacna1sa |  |  | 24037 | -0.395 | -0.2317 | No |
| 121 | 64612 | atp1a1a.1 |  |  | 24172 | -0.404 | -0.2321 | No |
| 122 | 407658 | acta1b |  |  | 24188 | -0.406 | -0.2287 | No |
| 123 | 569855 | cacng2b |  |  | 24247 | -0.410 | -0.2267 | No |
| 124 | 64613 | atp1a1a.2 |  |  | 24438 | -0.416 | -0.2287 | No |
| 125 | 100002384 | cox5b1 |  |  | 24457 | -0.417 | -0.2253 | No |
| 126 | 403310 | cacna1da |  |  | 24910 | -0.442 | -0.2354 | No |
| 127 | 415253 | zgc:86599 |  |  | 24928 | -0.443 | -0.2317 | No |
| 128 | 30616 | vmhc |  |  | 25400 | -0.475 | -0.2421 | Yes |
| 129 | 574004 | myl4 |  |  | 25467 | -0.481 | -0.2396 | Yes |
| 130 | 386711 | myh6 |  |  | 25613 | -0.495 | -0.2395 | Yes |
| 131 | 103911376 | si:ch211-226l4.6 |  |  | 25780 | -0.510 | -0.2399 | Yes |
| 132 | 393776 | cox4i2 |  |  | 25810 | -0.512 | -0.2359 | Yes |
| 133 | 494577 | cox6c |  |  | 25868 | -0.517 | -0.2328 | Yes |
| 134 | 100331199 | tnnt2b |  |  | 25902 | -0.520 | -0.2289 | Yes |
| 135 | 436768\_1 | uqcrq |  |  | 25910 | -0.521 | -0.2242 | Yes |
| 136 | novel.533 | - |  |  | 25954 | -0.525 | -0.2206 | Yes |
| 137 | 114410 | casq2 |  |  | 25956 | -0.526 | -0.2156 | Yes |
| 138 | 568512 | slc8a4a |  |  | 26080 | -0.536 | -0.2144 | Yes |
| 139 | 557348 | cox8a |  |  | 26159 | -0.544 | -0.2117 | Yes |
| 140 | 797409 | cacng5a |  |  | 26545 | -0.583 | -0.2183 | Yes |
| 141 | 337770 | si:ch73-187m15.4 |  |  | 26661 | -0.594 | -0.2163 | Yes |
| 142 | 58114 | actc1b |  |  | 26722 | -0.601 | -0.2125 | Yes |
| 143 | 565158 | cacnb3a |  |  | 26860 | -0.614 | -0.2110 | Yes |
| 144 | 436768 | uqcrq |  |  | 26944 | -0.624 | -0.2077 | Yes |
| 145 | 559964\_1 | cacna1fb |  |  | 27228 | -0.645 | -0.2105 | Yes |
| 146 | 564883 | cacng3b |  |  | 27260 | -0.646 | -0.2054 | Yes |
| 147 | 100333064 | uqcr10 |  |  | 27599 | -0.685 | -0.2095 | Yes |
| 148 | 768300 | vmhcl |  |  | 27872 | -0.725 | -0.2112 | Yes |
| 149 | 100329748 | LOC100329748 |  |  | 27955 | -0.737 | -0.2068 | Yes |
| 150 | 554154 | uqcrb |  |  | 28106 | -0.757 | -0.2044 | Yes |
| 151 | 100126126 | ryr2a |  |  | 28171 | -0.768 | -0.1991 | Yes |
| 152 | 100038781 | trdn |  |  | 28203 | -0.773 | -0.1928 | Yes |
| 153 | 415164\_1 | zgc:86725 |  |  | 28382 | -0.801 | -0.1908 | Yes |
| 154 | 570210\_1 | zgc:86709 |  |  | 28539 | -0.828 | -0.1879 | Yes |
| 155 | 415175 | tnnc1b |  |  | 28699 | -0.858 | -0.1847 | Yes |
| 156 | 556703 | tnnt2e |  |  | 28776 | -0.874 | -0.1788 | Yes |
| 157 | 58071 | tnnt2a |  |  | 29115 | -0.946 | -0.1805 | Yes |
| 158 | 335751 | cox7a3 |  |  | 29214 | -0.977 | -0.1744 | Yes |
| 159 | 100124599\_1 | smyhc2 |  |  | 29618 | -1.101 | -0.1766 | Yes |
| 160 | 64671 | cmlc1 |  |  | 29619 | -1.102 | -0.1662 | Yes |
| 161 | 100003220 | cacng6b |  |  | 29793 | -1.162 | -0.1606 | Yes |
| 162 | 794222 | cacng4b |  |  | 29968 | -1.231 | -0.1544 | Yes |
| 163 | 114457 | atp1b2b |  |  | 30050 | -1.265 | -0.1450 | Yes |
| 164 | 556638\_1 | slc9a6b |  |  | 30270 | -1.376 | -0.1388 | Yes |
| 165 | 550445 | acta1a |  |  | 30411 | -1.455 | -0.1294 | Yes |
| 166 | 100037358\_1 | zgc:163073 |  |  | 30530 | -1.526 | -0.1187 | Yes |
| 167 | 353247 | tnnc1a |  |  | 30580 | -1.568 | -0.1054 | Yes |
| 168 | 64609\_2 | atp1a2a |  |  | 30592 | -1.579 | -0.0907 | Yes |
| 169 | 64614 | atp1a1a.3 |  |  | 30843 | -1.802 | -0.0815 | Yes |
| 170 | 559015 | cacnb3b |  |  | 31158 | -2.162 | -0.0709 | Yes |
| 171 | 678560\_1 | cacnb4b |  |  | 31435 | -2.736 | -0.0537 | Yes |
| 172 | 393793\_1 | uqcrc1 |  |  | 31447 | -2.765 | -0.0278 | Yes |
| 173 | 447942\_1 | cox6a2 |  |  | 31777 | -4.401 | 0.0036 | Yes |
Table: GSEA details [plain text format]

  

Fig 2: CARDIAC\_MUSCLE\_CONTRACTION(DRE04260)      
 Blue-Pink O' Gram in the Space of the Analyzed GeneSet

  

Fig 3: CARDIAC\_MUSCLE\_CONTRACTION(DRE04260): Random ES distribution      
 Gene set null distribution of ES for **CARDIAC\_MUSCLE\_CONTRACTION(DRE04260)**
